# Supporting the well-being of new university teachers through teacher professional development

**DOI:** 10.3389/fpsyg.2022.866000

**Published:** 2022-07-28

**Authors:** Inken Gast, Madelief Neelen, Laurie Delnoij, Marloes Menten, Alexandra Mihai, Therese Grohnert

**Affiliations:** Department of Educational Research and Development, School of Business and Economics, Maastricht University, Maastricht, Netherlands

**Keywords:** psychological well-being, university teachers, professional development, faculty development, higher education

## Abstract

Over the last decades, changes within higher education have created increased pressure and uncertainty for academics, increasing their risk for cognitive, behavioral, physical, as well as psychological issues due to high job demands. Specifically, for new academics in teaching roles, their lack of knowledge and skills can contribute to a negative effect of these job demands on their well-being. This study therefore explored how teaching-related professional development programs can enhance new university teachers’ well-being, through semi-structured interviews with 10 university teachers participating in such a program at a mid-sized Dutch university. We pay special attention to the relationship between specific learning activities integrated in the program (such as learning communities, formal workshops, and reflecting) and various dimensions of the psychological model of well-being by Ryff and Keyes (such as self-acceptance, autonomy, environmental mastery, and positive relationships). Using co-occurrence analysis and content analysis, we found that different learning activities had distinct relationships with different well-being facets. For example, formal workshops were mainly related to environmental mastery, a purpose in life and personal growth, while reflecting seemed to be especially connected to teachers’ self-acceptance, and participating in a learning community was mainly related to positive relations with others and personal growth. Our findings have implications for research on teacher well-being as well as for the design of professional development programs for higher education teaching staff.

## Introduction

Academics are facing increasing pressure and uncertainty due to high workload for both teaching and research, along with changing performance expectations, which can negatively impact their overall well-being at work as well as their performance (Lackritz, 2004; Watts and Robertson, 2011; Shin and Jung, 2014). Academics’ work environment has become more stressful due to increasing pressure to publish and to acquire research funding in a highly competitive environment on the one hand, and through changes in education such as the digitalization of education (accelerated by the COVID-19 pandemic), increasing student numbers, and increasing personalization of support for students, on the other hand (Watts and Robertson, 2011; Kinman, 2014; Lei et al., 2021). Comparable to other service sector employees, academics are at high risk of experiencing burnout, occupational stress-related symptoms, diminished work satisfaction, and therefore, lower work well-being (Blix et al., 1994; Watts and Robertson, 2011; Kinman and Johnson, 2019), where employees with high work well-being are defined as flourishing employees who achieve their full potential (Schulte and Vainio, 2010). The human and financial costs attributable to dysfunctional employee psychological well-being have long been established, including cardiovascular diseases, long-term absence, and presenteeism (Diener, 1994; Salvagioni et al., 2017; Sabagh et al., 2018).

These concerns around well-being are exacerbated for new teaching staff, who lack the knowledge, skills, and attitudes (KSAs) to effectively manage such work demands (Hubbard and Atkins, 1995; Austin et al., 2007). New university teachers must navigate conflicting beliefs regarding their practice, which arise from professional norms, work culture, and personal expectations (Austin et al., 2007; Kessels, 2010), in environments where academics are often hired based on their academic achievements and not because of their teaching experience or interest in teaching (Morris and Usher, 2011; Graham, 2018). These new teachers are often not trained educators and have no formal teaching qualifications, which may result in low teaching efficacy (especially at the beginning of one’s teaching career), low motivation, stress, and ultimately negative effects on teaching performance and psychological well-being (Morris and Usher, 2011; Butcher and Stoncel, 2012; Stewart, 2014). Consequently, new university teachers require support for becoming effective teachers, as well as for fostering their well-being at work.

Programs or interventions focusing on employee well-being are often provided outside of work or are not embedded in participants’ daily work practice, which makes transfer of training to the workplace difficult (see Page and Vella-Brodrick, 2012; Karpavičiūtė and Macijauskienė, 2016). On the other hand, teacher professional development programs in higher education usually primarily focus on developing teachers’ KSAs (Mathieson, 2011; Gast et al., 2017; Fabriz et al., 2021) and are therefore closely related to and embedded in teachers’ work practice. However, the potential of teacher professional development programs for increasing teachers’ well-being at work is often overlooked (Hubbard et al., 1998). This study therefore explores how an established teaching focused professional development program can also be effective for improving teachers’ well-being. As professional development can take place through a variety of activities such as formal trainings or workshops (e.g., Steinert, 2010), informal learning on the job (e.g., Virolainen, 2007), learning in teams or through learning communities (e.g., Gast et al., 2017), or learning through individual reflection or experimentation (e.g., Clayton and Ash, 2005), this study takes a multi-faceted approach to understanding the link between development activities and university teachers’ well-being, in line with Acton and Glasgow (2015) and Onyura et al. (2017). Extant research within and outside of the university context has found that professional development empowers employees by providing both social and institutional support (Tansky and Cohen, 2001; Kraimer et al., 2011; Onyura et al., 2017; Heffernan and Heffernan, 2019). However, little is known to date about how specific teacher professional development activities are related to psychological well-being in the higher education context. Taking a case-study approach, this study aims to investigate how certain professional development activities in formal teacher professional development (e.g., workshops, communities of practice, and reflection) are related to university teachers’ well-being at work. The results of this study can inform the design of professional development programs or interventions that focus on the development of both KSAs and well-being of employees, in higher education as well as in other sectors.

### Teacher well-being at work

Although there is currently no consensus on the definition of work well-being, a commonly cited source described it as “flourishing employees achieving their full potential for both their own benefit and that of the organization” (see Schulte and Vainio, 2010, p. 423). In the literature, work well-being has been operationalized in various ways, for example, through the absence of ill-being, multidimensional models of well-being, job-related affective well-being, or by measuring work well-being through other related constructs such as job satisfaction, job involvement, work engagement, happiness at work, or organizational commitment (e.g., Page and Vella-Brodrick, 2009; Fischer, 2014; Bermejo-Toro et al., 2016; Branand and Nakamura, 2017). Because we see psychological well-being as the core of employee mental health, our study takes a multidimensional perspective on work well-being based on the model of psychological well-being developed by Ryff and Keyes (1995), and applies this model to the higher education work context.

According to their model of psychological well-being (Ryff and Keyes, 1995), well-being involves six dimensions: self-acceptance, positive relations with others, autonomy, environmental mastery, purpose in life, and personal growth. (1) The dimension of *self-acceptance* describes people’s positive attitude toward themselves, including their positive and their negative qualities. In the context of higher education, this relates to the development of a teacher identity (e.g., Van Lankveld et al., 2017) as well as teachers’ perception of themselves as active learners (Ryan and Deci, 2000). (2) The dimension of *positive and trusting relations with others* includes being concerned about and empathetic toward others. Within higher education, teachers have been shown to meet relatedness needs by building positive relationships with students, colleagues, and administrators (Split et al., 2011; Miles et al., 2015). (3) People who score high on the dimension of *autonomy* are described as independent and are able to resist social pressures. Autonomy is “most strongly felt by educators when they are free to make pedagogical decisions, rather than experiencing such decisions as being controlled externally (Averill and Major, 2020, p. 149).” (4) The dimension of *environmental mastery* describes people’s sense of competence in managing their environment and their ability to choose or create contexts that suit their needs and values. In higher education, environmental mastery is related to teachers’ development of their pedagogical and didactic skills, which provides teachers with the possibility of shaping their work environments inside and outside of the classroom in a way that best suits their strengths and beliefs (e.g., Wallace and Priestley, 2011). (5) *Purpose in life* describes people’s sense of directedness in life, where work-related goals create meaning for their life. Teachers’ purpose in life can manifest itself in the advancement of their career and in the sense of meaningfulness of their teaching role for student learning (Li, 2018; Rosewell and Ashwin, 2019). (6) Finally, the dimension of *personal growth* describes people having a sense of continued development and being open to new experiences. In higher education, this dimension can, for example, be related to teachers’ professional development, innovative work behavior and openness to experimentation (e.g., De Pablos-Pons et al., 2013). In this study, we take into account all six dimensions of well-being in studying their relationship with teacher professional development activities.

### Teacher professional development

Teacher professional development in higher education is a continuing process, as teaching takes place in an ill-structured and dynamic environment (Austin et al., 2007; Onyura et al., 2017; Larson et al., 2019). In this process, teachers are seen as life-long learners (Nicholls, 2000; Duţă and Rafailă, 2014) who participate in various activities ranging from formalized programs to informal learning at the workplace (e.g., Butcher and Stoncel, 2012; Gerken et al., 2016). Taking into account the broadness of the concept, teacher professional development can be defined as “a critical review process that allows training practice, contract reviews, learning which problems are faced by teachers, seeking solutions and building knowledge about the learning process” (Duţă and Rafailă, 2014, p. 803). As new university teachers usually do not have any prior teaching experience or educational training, teacher professional development programs, and postgraduate certificates in higher education teaching are common support structures to guarantee a high quality of education for students (e.g., Butcher and Stoncel, 2012; Stewart, 2014). To meet the needs of new teaching staff, these programs usually focus on a variety of teaching-related aspects such as pedagogy, course and curriculum design, student assessment, teacher beliefs, or teacher identity (Burton et al., 2005; Duţă and Rafailă, 2014).

Past research on the effectiveness of professional development programs for teachers in higher education have indeed provided robust evidence for a positive effect on teachers’ KSA development (e.g., Norton et al., 2010; Gerken et al., 2016; Phuong et al., 2018). Furthermore, studies by Green et al. (2013) and Nevgi and Löfström (2015) have shown that teacher professional development programs can help to shape academics’ teacher identity. In addition, prior research has shown that these programs should include a range of learning activities, such as collaborative and self-reflection, relationship building, feedback and experimentation (Norton et al., 2010; Onyura et al., 2017), as not all professional development activities are equally relevant for all teachers (Norton et al., 2010). Building further on this established link between professional development programs and teachers’ KSA development, this study explores the link between a variety of learning activities and teachers’ well-being.

### Professional development and psychological well-being of university teachers

We build our research question on the relationship between professional development activities and well-being by aligning Ryff and Keyes’ (1995) six dimensions of psychological well-being with state-of-the-art research on teacher professional development. First, we identify a series of professional development program outcomes that align with the dimension of *self-acceptance*: increased reflexivity, self-efficacy, and a clearer self-image and a stronger sense of their identity as an educator (Stewart, 2014; Onyura et al., 2017; Fabriz et al., 2021). We therefore expect that participating in a program with such outcomes has the capacity to foster new university teachers’ well-being through the dimension of self-acceptance.

Second, the dimension of *positive relations with others* is mirrored in teacher professional development research through providing social support at work, which by providing networking opportunities creates a feeling of increased social support, and supports forming affiliations for future collaboration (Mathieson, 2011; Onyura et al., 2017). Although many programs still have an individual focus, research has highlighted the benefits of participatory and collaborative learning (e.g., Stes et al., 2007; Warhurst, 2008). We therefore expect that teacher professional development programs can foster well-being when facilitating the making of social connections.

Third, previous research has shown that *autonomy* can be supported through professional development programs – for example, participants reported increased perceived credibility after participation in professional development activities that helped them to “develop a teaching voice in their departments” (Butcher and Stoncel, 2012, p. 156). In addition, studies have shown that professional development programs contribute to the empowerment of academics (e.g., Thorndyke et al., 2006). This link between professional development programs and autonomy further leads us to expect a positive link with well-being.

Fourth, the dimension of *environmental mastery* aligns with teacher professional development research through the development of KSAs, which enables teachers to adapt and innovate their teaching practices and to create a work environment that fits their strengths and beliefs (see Butcher and Stoncel, 2012; Onyura et al., 2017). When the teaching values that participants develop during these programs align with those promoted by their department, transfer from training to practice is likely (Stes et al., 2007), providing a condition for how teacher professional development programs can positively contribute to new teacher well-being.

Fifth, teacher professional development programs can contribute to an increased sense of *purpose in life*, as they can help participants to find meaning in their teaching roles (Onyura et al., 2017). Teachers who can engage in their work with increased passion will likely also experience a sense of purpose (Yukhymenko-Lescroart and Sharma, 2019). Furthermore, teachers in Butcher and Stoncel’s (2012) study reported being more engaged in their department’s teaching-related discussions after completion of a professional development program. These findings support our view that by creating purpose in life, teacher professional development programs can foster well-being.

Finally, related to the dimension of *personal growth*, participants in past research have often reported a willingness to try new teaching approaches (Butcher and Stoncel, 2012; Stewart, 2014). Stewart (2014) described a shift in participants’ view of the professional development activities from a task-oriented to a more meaning-oriented view, which was related to participants’ increased self-esteem. In sum, therefore, we expect a positive link between teacher professional development programs and all six facets of psychological well-being.

Overall, the current literature provides a general view of how teacher professional development has the potential to support university teachers’ well-being. However, most of the literature has concentrated on how a general teacher professional development program affects teaching performance and student learning. Consequently, these studies provide only limited information about whether and how specific aspects of the program play a role in teachers’ psychological well-being. By taking a case study approach, our study therefore aims to explore how specific activities of a teacher professional development program relate to the psychological well-being of new higher education teachers.

### The case: A Dutch teacher professional development program

Our study was conducted in the Business and Economics faculty at a mid-sized Dutch university adopting student-centered pedagogy. In the Netherlands, all higher education teaching staff are required to complete a formal teacher professional development program to obtain an official higher education teaching qualification (University Teacher Qualification, UTQ). At all Dutch universities, this UTQ program focuses on the same basic teaching competences related to course and curriculum design, teaching delivery, student assessment, and organization of education. These competences are connected to the course design principles of constructive alignment (Biggs, 1996; Biggs and Tang, 2015), with the goal of ensuring the quality of the educational program. However, all universities tailor this program to the specific needs of their teachers, for example, based on differences in pedagogy. The UTQ program aims to support teachers in the development of teaching KSAs and vision necessary to teach at a university in the Netherlands (see Universiteiten van Nederland, 2022).

**FIGURE 1:**
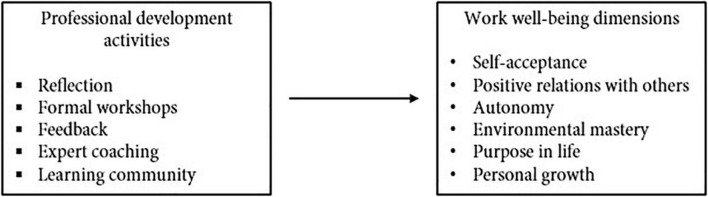
Research model.

In our case study, the professional development program consisted of three phases: self-assessment, competence development, and assessment. The program included various professional development activities connected with the main teaching competences targeted by the program, which involved reflection, interactive workshops, feedback, coaching and a learning community. This case study therefore enabled us to study the link between five types of professional development activity and the six dimensions of psychological well-being, as illustrated in Figure 1.

#### Reflection

Reflection is defined as “the process or means by which an experience, in the form of thought, feeling, or action, is examined to distil its meaning while it is happening or subsequently” (Scott, 2010, p. 432). Through reflection in action, teachers can revisit a sequence of events and the reactions involved; the individual is reflecting on a previous experience (Yanow and Tsoukas, 2009). In the current case, participants engaged in reflection through self-assessment and a reflective portfolio, which encouraged ongoing reflection while implementing new teaching methods. In the initial phase of the program, participants were asked to reflect on the targeted teaching competences using a self-assessment form. Based on this self-assessment, participants set learning goals for their competence development in the program and drew up an action plan on how to achieve these goals. In the program’s competence development phase, participants wrote a reflective portfolio in which they reflected on their course design, teaching practices, student assessment and overall professional development.

#### Interactive workshops

Workshops within teacher professional development program often focus on pedagogical knowledge or subject-related content knowledge (Mishra and Koehler, 2006), social and affective topics (Taylor and Berry, 2008), communication, conflict management, student motivation (Duţă and Rafailă, 2014), or the exchange of best practices (Taylor and Berry, 2008). The overall goals are to create a style of teaching that is personal, but also aligned with the goals and mission of the university, improve teachers’ KSAs and increase teachers’ professional confidence (e.g., Hanbury et al., 2008). In the case study at hand, workshops provided participants with the relevant pedagogical knowledge for each target competence. They were highly interactive and focused on the improvement of participants’ own courses by setting course objectives, choosing appropriate teaching methods, developing educational materials, student-centered teaching and communication, student formative and summative assessment, peer assessment, and administrative embedding.

#### Feedback

Feedback is valuable to professionals because it helps them to keep their performance up to or above organizational standards (Sparr and Sonnentag, 2008). This includes both supervisor feedback for role clarity, which helps to enhance competences, reduces uncertainty regarding goal-related behavior, and control (in line with Ilgen et al., 1979; Ashford and Cummings, 1983), and peer feedback for addressing common challenges and learning from each other (Huston and Weaver, 2008). Feedback on teaching behavior can become a valuable informational resource to achieve personal teaching goals (Kluger and DeNisi, 1996), as it relates to both “the appropriate behaviors to achieve a goal (referent information) and how well an individual is enacting those behaviors (appraisal information)” (Ashford and Cummings, 1983, p. 372). In the current case study, informal learning from feedback partially overlapped with formal learning in workshops, as feedback sessions were included in workshop activities. Teachers received peer feedback during the workshops, as well as feedback from an educational expert on their reflective portfolio. Finally, an educational expert observed one of their classes and provided feedback on their teaching behavior.

#### Expert coaching

Coaching from a more experienced colleague can serve learning, directive, supportive and motivational functions in a professional development program (e.g., Theeboom et al., 2014). Coaching has been shown to influence not only employees’ self-conceptions and performance, but also employee well-being, through reducing occupational stress (Grant et al., 2009). In the UTQ program, coaching was provided by educational experts in the context of and beyond workshops whenever the participants needed support or guidance. Coaching was a voluntary activity in the program, and participants could ask for individual coaching whenever necessary.

#### Learning community

The importance of understanding how teachers work together and share practices is reflected in the large number of studies looking at teacher teams, communities of learning, and peer coaching (Onyura et al., 2017). In higher education, learning communities can foster collaborative work cultures for teachers by promoting and sustaining the learning of teachers with the purpose of enhancing student learning (Vescio et al., 2008). This view assumes that knowledge is situated in the day-to-day experiences of teachers and best understood through critical reflection with others who share the same experiences (Hairon and Dimmock, 2012). Additionally, professional learning communities also facilitate information sharing and psychological support (Lieberman, 2000). Onyura et al. (2017) found that professional development programs create a platform for the establishment of cross-professional and cross-organizational collegial relationships, which result in support networks and collaborative initiatives. Within the UTQ program, participants were organized in cohorts within faculties. They went through the second program stage together and were expected to collaborate on course design and teaching delivery tasks. During the interactive workshops, an experienced colleague or educational expert leveraged their knowledge with empirical evidence and facilitated the exchange and challenge of beliefs and values. Additional voluntary community activities were provided, such as writing up meetings for the reflective portfolio.

## Methodology

### Qualitative case study

To answer the research question, a qualitative research approach involving a qualitative case study design was chosen. The qualitative research approach made it possible to study the nature of the relationship between specific formal teacher professional development activities and university teachers’ well-being with more depth, taking into account the perceptions of the teachers themselves (Fossey et al., 2002; Merriam and Tisdell, 2016). The chosen case study design allows for the in-depth study of this relationship in the specific context (Meredith, 1998) of the previously described UTQ program as implemented in a specific faculty at a Dutch university. The studied phenomenon is intrinsically bounded (Merriam and Tisdell, 2016) as it is limited to a small number of university teachers participating in the specific implementation of the UTQ program.

### Semi-structured interviews

Semi-structured interviews were conducted with university teachers currently participating in the aforementioned teacher professional development program. Semi-structured interviews start with a well-developed set of questions (interview guide) while at the same time leaving sufficient room for participants to add information where necessary [in line with practices of Jackson et al. (2007; see Table 2)]. This approach was chosen to allow for deeper insights into participants’ perceptions (Jackson et al., 2007) as well as to allow structure and comparability of the findings while also giving freedom to the interviewer and interviewee to explore certain topics in more depth, if desirable (Yin, 2003; Jackson et al., 2007). Through these semi-structured interviews, we could explore in-depth the relationships between the teacher professional development activities and the well-being dimensions.

**TABLE 1:** Overview of interviewees’ demographics.

Demographics		*N* = 10
Gender identity	Female	6
	Male	4
Age	33–42 years	4
	27–32 years	6
Educational level	Master’s	4
	Ph.D.	6
Position	Ph.D. candidate	3
	Lecturer	1
	*Post doc* researcher	1
	Researcher	1
	Assistant professor	4
Contracted teaching	20/80	4
load (%	40/60	1
teaching/research	50/50	4
distribution)	100/0	1
Years teaching at	1–4 years	7
faculty	5–10 years	3

**TABLE 2:** Coding scheme and example interview questions and coded segments.

Category name	Definition	Example interview questions	Example coded segment
**Psychological well-being**			
Self-acceptance	Possesses a positive attitude toward the self; acknowledges and accepts multiple aspects of self, including good and bad qualities; feels positive about past life (Ryff and Keyes, 1995)	Following the program, how has your perception changed about how you feel about your achievements as a teacher?	“It did help me in making me realize what competences are important as a teacher and that made me even more aware of how I am as a teacher while I was teaching as well. I became more conscious […] and learned to be more vulnerable, to be more authentic.”
Personal growth	Has a feeling of continued development, sees self as growing and expanding, is open to new experiences, has sense of realizing his or her potential, sees improvement in self and behavior over time, is changing in ways that reflect more self-knowledge and effectiveness (Ryff and Keyes, 1995).	How do you think the program helped in identifying and setting your own targets for development?	“It showed me that I need to develop myself as a teacher, more in what I do as a teacher, and how I would implement it, how I would act as a teacher, what I need to do as a teacher in order to be effective, I am a little bit more intentional about what I am doing, what I want to achieve, what I can do differently.”
Autonomy	Is self-determining and independent, able to resist social pressures, to think and act in certain ways, regulates behavior from within, evaluates self by personal standards (Ryff and Keyes, 1995).	How do you feel due to the program about making your own teaching-related decisions?	“For tips and tricks, all the examples you heard from your peers are now in the back of your mind, where you can each time think is it something that might work for your course or not, there are more options now that are available for you, many more than you individually had at the start.”
Purpose in life	Has goals in life and a sense of directedness, feels there is meaning to present and past life, holds beliefs that give life purpose, has aims and objectives for living (Ryff and Keyes, 1995).	How does the program influence your life in terms of thinking about the future as an academic?	“The importance of aligning and matching it up has been made more prominent by the program. The purpose as a teacher, that refers back to the active engagement role, how I evolved and can still evolve on how I can play a role in stimulating students.”
Positive relations with others	Has warm, satisfying, trusting relationships with others, is concerned about the welfare of others, is capable of strong empathy, affection, and intimacy, understands the give and take of human relationships (Ryff and Keyes, 1995).	What is the role of the program in how you maintain close work relationships and share your time with colleagues?	“One new colleague participated in one workshop, we kind of talked about how to go about those things. Afterward she said it was very helpful that we discussed this and this. And I asked her if she learned something from this particular period. Like yes, I am going to do this and this next period.”
Environmental mastery	Has a sense of mastery and competence in managing the environment, controls a complex array of external activities, makes effective use of surrounding opportunities, able to choose or create contexts suitable to personal needs and values (Ryff and Keyes, 1995).	How has the program played a role in managing the responsibilities of daily work life?	“It really made me think about what is useful, but also how I can use that on a daily basis, it gives me more structure on how I approach teaching or designing a course, in terms of thinking what I have to do, what the steps are, where do I start and what is the end goal, I think that is very helpful, anything that is practical I really enjoyed those and it gives tools, you can directly use the information for courses.”
**Professional development activities**			
The program in general	A learning process, resulting from meaningful interaction between the teacher and the professional context (Kelchtermans, 2009).	–	“A program where you learn to be conscious, you are learning tools to use during class, the didactics. By getting to know peers and sharing experiences you get an insight in how it’s done in class by others and how you do that and it also provides a more general overview of how education is built up so the courses do not only ask what is happening in class but also how it is organized.”
Reflection	Reflecting in the portfolio on the developed educational materials, the teaching delivered and the feedback, to improve teaching based on these reflections; reflecting on performance as a teacher to continually develop in this role based on these reflections by setting goals (Van de Wiel et al., 2018)	Do you think reflection has changed your perspective and pedagogical approach on teaching and learning?	“Reflection is the starting point and it is the key element of learning to realize that you have to improve or did something great, something that you will keep up in the future, and also to be critical with yourself mostly with the formal process, and evaluation is the most important part.”
Formal workshops	Working on one’s own courses, improvement of educational materials, conveying knowledge and practicing professional skills; learning to organize and plan the development of educational materials, assessment, administrative embedding, and the finalization of teaching activities (Van de Wiel et al., 2018).	How do you perceive the purpose and effectiveness of the formal workshops?	“Getting some education in how to educate others and how to design education at a higher education level. Thinking about assessment, learning about all the procedures and regulations that come with teaching at a university and specifically at this university, you also learn about [didactic] methods, the cognitive effects of learning on the learners.”
Feedback	Can relate to the appropriate behaviors to achieve a goal (referent information) and to how well an individual is enacting those behaviors (appraisal information) (Ashford and Cummings, 1983, p. 372).	Has the feedback given you the tools to review your own professional development needs?	“A couple of workshops I attended where my bachelor course was used as an example and I am quite proud to say this, quite good example as well. People used this as inspiration for their own courses and I received useful questions and feedback afterward.”
Expert coaching	Serves learning, directive, supportive and motivational functions in a professional development program as mental support (Ashford and Cummings, 1983).	How do you perceive the purpose and effectiveness of the coaching?	“Giving you advice on things, mainly for you to know, who to contact in certain situations […] from those people I learned a lot in regard to teaching, by asking questions, by discussing the issues I encountered during teaching.”
Learning community	Working together with colleagues to develop and coordinate teaching activities, discussing and contributing actively to knowledge exchanges and sharing experiences, learning from each other (Van de Wiel et al., 2018).	What is the role of collegial discussions with peers in the program?	“I think it’s more exchanging experiences with other teachers that is useful in some ways, or more how to handle conflict, given that you see other people, because you get various insights or ideas you get that you can then experiment or integrate in your course.”

### Sample

In total, 10 university teachers (see Table 1 for an overview of demographic characteristics) from six different departments within the business and economics faculty voluntarily participated in the interviews. The purposive sampling method was used to select the sample of participants among all the participants of the UTQ program. At the time of the interview, participants had to be either active participants of the UTQ program for at least a year or recent graduates. By applying these sampling criteria, it was possible to ensure that participants had participated in the program long enough to experience all different professional development activities of the program. Four participants identified as female and six participants identified as male. The age of the participants ranged from 28 to 43 years old, with an average age of 32.5 years (*SD* = 5.04). Furthermore, participants were teaching at the faculty for 3.4 years (*SD* = 2.58), on average. All participants were untenured junior faculty, for example, PhDs, junior lecturers, and assistant professors, because the teacher professional development program is aimed at new university teachers. Only one participant was a full-time teacher; the other participants had a contracted teaching load of between 20% and 50% of their time.

### Data collection procedure

The semi-structured interviews were conducted online, as face-to-face interviews were not feasible due to the COVID-19 pandemic regulations in the Netherland at the time. Participants were contacted *via* email and received a general outline of the study. Before participation, each participant signed an informed consent form agreeing to audio-taping of the interview, the anonymous reporting of findings for research purposes, and acknowledging their right to withdraw from participation at any moment without the need to give a reason. The interviews were conducted in English, the working language for teaching in the chosen faculty. The average interview took 58 min, with a range of between 52 and 69 min. Before the interviews took place, a pilot interview was conducted to test the interview guide.

### Interview guideline

The interview guide was based on the theoretical framework and focused on the six dimensions of psychological well-being (Ryff and Keyes, 1995), as well the described teacher professional development activities (based on Norton et al., 2010; Butcher and Stoncel, 2012; März and Kelchtermans, 2013). One or two main questions were asked per topic, followed by additional probes (see Table 2 for the coding scheme). The questions were open-ended to ensure in-depth discussion of the topics (Hsieh and Shannon, 2005).

### Coding procedure

All interviews were transcribed verbatim. The unit of analysis was meaningful segments within the interviewee’s response (Flick, 2014). These segments were of different lengths, ranging from part of a sentence to a set of related sentences. Thematic coding was applied to identify segments that were connected by a common theme, allowing for their assignment to categories (Braun and Clarke, 2012). Categories were defined based on the professional development activities of the teacher professional development program and the six dimensions of psychological well-being described by Ryff and Keyes (1995; see Table 2). Therefore, the interviews were mainly coded deductively, based on theoretical constructs of interest identified in the theoretical framework. Only one inductive code was added, regarding the role of the teacher professional development program as a whole, instead of focusing on specific teacher professional development activities.

Inter-coder reliability was checked by asking an independent second coder to assess a sample of 10% of meaningful segments (Poortman and Schildkamp, 2012). Any differences in coding were discussed by both coders and recoding was performed where necessary. Krippendorff’s c-Alpha Binary index was used to evaluate the inter-coder reliability, as it is applicable for small sample sizes and therefore suitable for our study design, ranging from 1 (perfect agreement) to 0 (perfect disagreement; Krippendorff, 2004; LeBreton and Senter, 2008). In our study, the total agreement coefficient yielded an index of 0.851, a satisfying result according to Krippendorff (2004): “it is customary to require α ≥ 0.800, where tentative conclusions are still acceptable, α ≥ 0.667 as the lowest conceivable limit” (p. 429).

### Data analysis

Qualitative data analysis software (ATLAS.ti, version 9.1.7.0) was used for the coding and analysis of the data. Directive content analysis was conducted by applying the deductive codes to the interview transcripts (Hsieh and Shannon, 2005). Along with in-depth qualitative analysis of the data, ATLAS.ti was used to conduct co-occurrence analysis between the codes for the six dimensions of psychological well-being on the one hand, and the five professional development activities on the other.

### Quality criteria

To increase the reliability and validity of the study, the quality criteria for qualitative research as described by Poortman and Schildkamp (2012) were followed as closely as possible. For example, a research design was chosen that fit the nature of the research question. Furthermore, the interviews were audio taped and analyzed by qualitative data analysis software. Data collection and data analysis were conducted in a structured manner creating a ‘chain of evidence.’ A clear description of the research steps and sampling strategy was provided. Multiple researchers were involved in the study and a good inter-rater reliability (Krippendorff’s c-Alpha Binary index of 0.851) between coders was established. Finally, the findings were interpreted in relation to prior theory.

## Results

Using co-occurrence analysis, we explored the link between the six professional development activities of the UTQ program (reflection, interactive workshops, feedback, expert coaching, learning community, and the program overall) and the six dimensions of psychological well-being (self-acceptance, positive relations with others, autonomy, environmental mastery, purpose in life, and personal growth). Table 3 reports the co-occurrences between these professional development activities and well-being, including overlapping and adjacent codes.

**TABLE 3:** Co-occurrence table of the professional development activities and psychological well-being dimensions.

		Professional development activities						
		Reflection	Formal workshops	Feedback	Expert coaching	Learning community	The UTQ program	Total
Work well-being dimensions	Self-acceptance	**8** (19)	**4** (4)	**1** (3)	–	**4** (5)	**1** (1)	32
	Positive relations with others	–	**2** (3)	–	–	**8** (17)	–	20
	Autonomy	–	**3** (4)	**1** (1)	**1** (1)	**5** (9)	**2** (2)	18
	Environmental mastery	**1** (1)	**10** (33)	–	**3** (3)	**5** (7)	**1** (1)	46
	Purpose in life	**4** (7)	**6** (9)	–	**2** (3)	**2** (2)	**5** (12)	33
	Personal growth	**4** (4)	**6** (12)	**5** (5)	**4** (6)	**9** (15)	**2** (2)	43
	Total	31	67	12	13	56	18	

In each cell, the number of participants mentioning a co-occurrence (*n*) is displayed in bold followed by the number of co-occurrences (*c*) in brackets. The colors represent the number of participants mentioning a co-occurrences dark gray (*n* ≥ 5), light gray (0 < *n* < 5), and white (*n* = 0).

**FIGURE 2:**
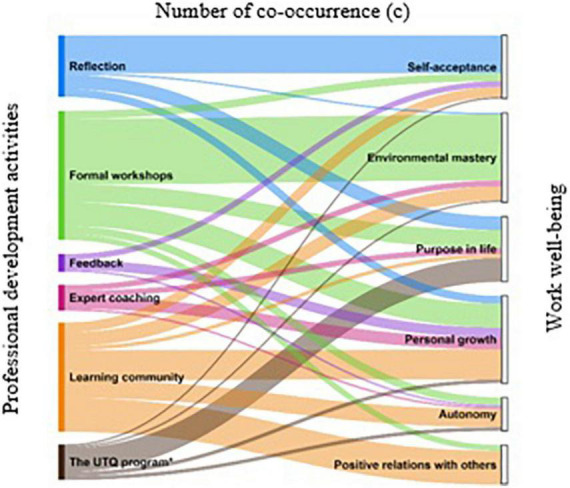
Sankey diagram of the number of co-occurrences. *The UTQ program contains all other professional development activities (reflection, formal workshops, feedback, expert coaching, and the learning community).

Table 3 provides an overview of the number of participants (*n*) mentioning a co-occurrence, and the number of co-occurrences (*c*) found. The psychological well-being dimensions of environmental mastery (*c* = 46) and personal growth (*c* = 43) were most frequently associated with the teacher professional development activities. The two dimensions of well-being that were least frequently associated with the professional development activities were positive relations with others (*c* = 20) and autonomy (*c* = 18). Comparing the professional development activities, formal workshops (*c* = 67) were associated most often with the dimensions of well-being, followed by learning community (*c* = 56). Both activities, along with the general UTQ program, were the only activities that were associated with all six psychological well-being dimensions. Expert coaching (*c* = 13) and feedback (*c* = 12) were least often associated with the dimensions of psychological well-being. For a visual representation of the co-occurrences between the professional development activities and the six dimensions of work well-being, see Figure 2. We discuss the co-occurrences in more detail for each well-being dimension below.

### Self-acceptance

The self-acceptance dimension of psychological well-being at work was most often associated with the professional development activity of *reflection* (*n* = 8, *c* = 19). During the interviews, reflection was described as an activity that gave participants the opportunity to acknowledge and accept both positive and negative aspects of their teaching practices and behaviors, which helped them to create a positive attitude toward themselves: “I think it is a good way to critically look at your own strengths and deficiencies” (Interviewee C). The self-assessment questionnaire at the beginning of the program helped participants to reflect on their teaching practices in a formal way and helped them to realize their tacit teaching practices:

It showed me that while intuitively I do a lot of things correctly […], it is great in formalizing a lot of the things that you often intuitively do as a teacher, it gives you a clearer explanation of why you do certain things, or why you have to do certain things. (Interviewee B).

Reflection also helped participants to realize what skills they were still lacking: “The first time I reflected on these competences, I realized that this is something I haven’t worked on” (Interviewee E).

Most other UTQ activities were mentioned less frequently in connection with self-acceptance. The *learning community* was associated with self-acceptance in four interviews (*c* = 5), where participants described that sharing experiences with colleagues helped to create a positive attitude toward themselves, by finding out that they were not the only one struggling with some teaching-related aspects:

Through discussions, you share a certain issue that you experience […] I don’t feel like an imposter anymore […] peers come in to help you to realize that you are not alone, and you are all learning how to become good teachers. (Interviewee B).

Furthermore, seeing different teaching styles in the community helps participants to find and accept their teacher identity: “You feel part of a collective of teachers, even though there are vastly different kind of teachers […] that affected my identity as a teacher” (Interviewee I).

*Formal workshops* were mentioned four times in connection with an increased self-acceptance (*n* = 4). The participants described how workshops fostered the insight that teaching came naturally for them, but they also confirmed what pedagogical knowledge they still needed to gain. Expert coaching was not associated with self-acceptance.

*Feedback* was only associated with self-acceptance in one interview (*c* = 3). For example, teachers received feedback from more experienced colleagues in the program, which helped them to acknowledge points for improvement and recognize the positive aspects of their teaching practices, and increased their positive self-perception. One participant described how the overall *UTQ program* (*c* = 2), helps them to gain more trust in themselves: “I now feel more justified about feeling good, because of the UTQ program, I know I did some stuff really well, I can appreciate more if I did a good job, because I know why it is good” (Interviewee C).

### Positive relations with others

The well-being dimension of positive relations with others was mainly associated with the *learning community* (*n* = 8, *c* = 17) created within the UTQ program. Going through the program together for a longer period of time helped to foster social connections among the participants. They got to know each other, their teaching practices, their struggles, and created new connections because of that. The possibility of building relationships with colleagues across various departments with whom you usually do not get into contact was especially highlighted: “Getting to know each other cross-departmentally, you can share research insights, which otherwise wouldn’t take place, because this is the only place where you meet” (Interviewee J). These new connections also created opportunities for collaborations beyond the UTQ program itself, on a professional level as well as on a personal level:

One colleague, we met afterward at the gym and we discovered that we are almost neighbors, it helped socialization in that sense. I met another colleague, we discovered that we have a common interest in research, I was invited to one of his lectures, and I invited him. (Interviewee G).

Additionally, the learning community strengthened already existing relationships.

The exchange of experiences and issues on a deeper level during the *formal workshops* (*n* = 2, *c* = 3) helped to create relationships between the participants: “The workshops are great because you learn from each other” (Interviewee B). Likewise, the theoretical frameworks discussed in the workshops provided a base for advanced and empathetic discussions outside of the workshops. Due to the discussions during the workshops, participants started to relate more to their colleagues: “You realize, you have been in the same situation” (Interviewee B).

*Feedback*, r*eflection*, *expert coaching* and the *UTQ program* in general were not associated with the dimension of positive relations with others.

### Autonomy

The well-being dimension of autonomy, although associated with almost all professional development activities, was only occasionally mentioned in the interviews. Autonomy was most often associated with the *learning community* (*n* = 5, *c* = 9). The learning community was said to open up room for active discussions supporting the participants in becoming more able to resist social pressures and evaluate whether a teaching method suggested to them actually fit within their course. Furthermore, the learning community encouraged learning from others, while also strengthening self-determination of one’s own teaching practices: “I know when people say that teaching is not important, I know for myself it has a certain importance and standing for me that I will put a certain effort into it” (Interviewee B).

*Formal workshops* (*n* = 3, *c* = 4) helped participants gain confidence through increased competence, which also increased their self-determination. In addition, the workshops helped participants to make autonomous decisions: “If I would be a coordinator, I would still follow my own gut, the meeting helped in crystallizing arguments contra and in favor of it, you really in the end learn the pros and cons of it” (Interviewee I).

Participants described how the *UTQ program* as a whole helped them to feel less dependent on others (*n* = 2, *c* = 2):

Due to the UTQ program, I will be better in handling it myself, although you will always have discussions with your peers, in the future I will have to do things myself, now I feel like I am much better prepared to do it by myself as a teacher. (Interviewee B).

The availability and help of *expert coaches* (*c* = 1) supported one participant in feeling more self-determined and confident, and able to coordinate a course independently. *Feedback* was also only associated once with the dimension of autonomy in connection with the participant’s feeling of increased autonomy due to concrete solutions that were discussed regarding the assessment in their course. *Reflection* was not associated with the dimension of autonomy.

### Environmental mastery

All 10 participants in the UTQ program associated environmental mastery with the *formal workshops* (*c* = 33). In general, the workshops supported participants in acquiring important teaching-related knowledge which they needed to effectively design courses and, thereby shape their work environment in a way that is effective for student learning, but also fits their own teaching style and personal needs. The workshops also supported the participants in learning how to collaborate effectively with colleagues, and participants felt responsible for conveying their newly acquired knowledge to their colleagues as well:

I feel responsible to make sure that [what I learned] from my UTQ courses somehow gets through to them in their tutorials. I am really working on the tutor manual, I have tutor meetings where I tell them they should approach it in this way, the UTQ program is definitely helping me in that process. (Interviewee C).

Moreover, workshops that provided practical information about administration and regulations provided concrete tools to effectively make use of surrounding opportunities. Similarly, the workshops supported participants’ efficiency and time management: “It made me more aware of the different roles, how much work and time you can be expecting to put into it, now after the workshops, I am able to assign different amounts of time to those roles” (Interviewee E).

Being part of a *learning community* (*n* = 5, *c* = 7) also contributed to participants’ environmental mastery through sharing of experiences and ideas. One participant, for example, mentioned that the discussion of specific cases helped them feel prepared to manage a complex array of teaching activities. Discussions with peers also helped participants to learn how to work together effectively with colleagues in a teaching team. By coordinating certain teaching activities with others, another respondent felt able to create contexts suitable to their personal needs and values. For example, they learned how to better organize their teaching teams, especially when working in a large team.

*Expert coaching* (*n* = 3, *c* = 3) helped participants by providing guidance on how to approach certain teaching activities. One participant also mentioned that coaching helped them to support the professional development of one of the tutors they were working with:

One of my tutors didn’t perform well, he scored really badly with students, so I asked the [coach] for a personal meeting to fix and discuss it also with my tutor […] she offered us some solutions, some workshops that my tutors could attend. (Interviewee G).

Moreover, expert coaching also provided mental support, helping to create contexts suitable for the personal needs of this interviewee:

It also comforts me, that there is such a support system in the form of the [coach] for the UTQ program but also in the form of other colleagues who are providing the workshops and I know that you can easily ask them for advice, help, or feedback. (Interviewee E).

Reflection and the UTQ program in general were both only mentioned once in relation to the dimension of environmental mastery. *Reflection* stimulated the interviewee to evaluate their courses more often and to create contexts such as teaching methods suitable for their personal values and needs. The *UTQ program* in general was associated with increased environmental mastery in relation to the usefulness of the program regarding the management of administrative tasks and regulations. Finally, *feedback* was not associated with environmental mastery.

### Purpose in life

The well-being dimension of purpose in life was mainly associated with the *UTQ program* (*n* = 5, *c* = 12) in its entirety. Interviewees described how participation in the program had shaped their academic identity: “Being employed at a university, teaching is paramount and makes a big part of why you are there” (Interviewee B). Furthermore, the UTQ program helped the participants to identify themselves as part of a bigger teaching community: “I had a realization moment, it is not only my course, it is part of a whole program, a whole year, a whole faculty” (Interviewee I). In addition, the UTQ program opened up new career opportunities for people, such as a teaching career or a tenured position.

*Formal workshops* (*n* = 6, *c* = 9) contributed to participants’ sense of purpose in life by giving direction to their teaching activities and by helping to them to better support student learning. Furthermore, these workshops provided clarity to teachers regarding what is expected of them, and helped them to design their teaching activities accordingly. Finally, participants explained that the workshops helped them to understand the reasoning behind certain teaching practices, helping them to make informed choices to reach their teaching goals: “I feel at least the decisions that I make are not so much from intuition or personal belief, actually I now have more arguments that I can use, based on the program, […] research shows this and this” (Interviewee H).

*Reflection* (*n* = 4, *c* = 7) also contributed to participants’ sense of purpose in life. By developing their personal reflective portfolio, participants felt that they were ready to incorporate what they had learned in their future teaching roles. Reflection helped the participants evaluate how the acquired teaching competences contributed to their teaching career. Furthermore, one interviewee mentioned that reflections helped to create meaning and a sense of directedness to continue with their usual teaching practices: “It’s like a confirmation [for] the teaching that I did, the way that makes me feel, it makes me so happy, that I want to continue with that” (Interviewee H).

Expert coaching and the learning community were only seldom associated with purpose in life. Participants reported that *expert coaching* (*n* = 2, *c* = 3) helped them to set and talk about their goals related to their teaching practice. Furthermore, a coaching meeting at the end of the program was described by a recent graduate as helpful when setting goals for their future professional development as a teacher. The *learning community* (*n* = 2, *c* = 2) also helped participants to set teaching goals by creating an opportunity for the participants to share experiences: “Through discussions you just realize that we all face some of the same issues […] through sharing of knowledge you are able to implement it, or think about how you would think about it in the future” (Interviewee B). In addition, the feeling of being part of a community supported respondents in the feeling that there is meaning to their teaching role. *Feedback* was not associated with the dimension of purpose in life.

### Personal growth

The well-being dimension of personal growth was most often associated with the learning community as well as the formal workshops in the UTQ program. Almost all interviewees associated the *learning community* (*n* = 9, *c* = 15) with their personal growth as a teacher. Discussing issues and contributing actively toward discussions increased learning from each other and stimulated continual development. Participants described how they improved their teaching methods based on the ideas of others. Furthermore, the exchange with peers helped participants be more open to new experiences:

A lot of new ideas pop up which makes you immediately more open to thinking and reflecting about those options yourself. Otherwise, you remain stuck in what you have been doing yourself for a while already and you are not really exposed to other things. (Interviewee D).

Several interviewees mentioned that they gained theoretical and practical knowledge during *formal workshops* (*n* = 6, *c* = 12) and saw themselves growing and expanding as a teacher over time: “You gain more knowledge on how to put everything into practice, you constantly assess all the things you pick up in the workshops to apply to your own situation, such that you constantly improve your teaching activities” (Interviewee D). Furthermore, participants reported that they had become open to new experiences and saw themselves growing as a result. They were also experimenting more with new teaching and assessment methods: “I am experimenting more with the assessment methods and aligning those, […] the program was very helpful in that” (Interviewee E).

The interviewees mainly mentioned *feedback* (*n* = 5, *c* = 5) received from experts and peers during the workshops in association with personal growth. The feedback was followed up and incorporated in order to expand their teaching skills: “One of my colleagues was there [during the tutorial] to give me feedback, […] afterward we had an evaluation about how I could do it differently next time […] this is a critical incident where I learned a lot” (Interviewee H).

*Expert coaching* (*n* = 4, *c* = 6) especially served a directive and learning function for the interviewees, and helped with improving their behavior over time. For example, one teacher received expert coaching when struggling with a specific tutorial group:

This is not how I would like to have it, and I thought, how can I change that, then I went to the [coach] to ask some tips how to deal with that […] Suddenly, it went really well in that group so that was a really good intervention. (Interviewee H).

Participants also reported that expert coaching helped them to realize their potential. *Reflection* (*n* = 4, *c* = 4) helped participants to become more self-critical and see improvement over time. Similarly, another interviewee stated that the reflective portfolio had helped them to gain more insight into what decisions they made and how these helped them to change their behavior over time. The *UTQ program* (*n* = 2, *c* = 2) as a whole helped participants to be open to new experiences, displaying a sense of personal growth.

## Conclusion and discussion

The aim of this study was to investigate how certain professional development activities in a formal teacher professional development program are related to new university teachers’ well-being at work. The results of our case study show an overall link between professional development activities and well-being at work, with specific activities playing a larger role for university teachers’ well-being than others.

First, in our case study, the well-being dimension of self-acceptance was mainly related to reflection, with participants emphasizing that reflection helped them acknowledge and build on their strengths and weaknesses as a teacher. In the field of education, reflective practice has previously been linked to a range of outcomes that facilitate self-acceptance, including increased status and self-esteem (Beck and Kosnik, 2001), as well as self-efficacy from reflecting on positive and negative aspects of one’s teaching (Yost, 2006). In addition, reflection supports the psychological well-being of academics because they acknowledge a renewed sense of their identity as an educator (März and Kelchtermans, 2013; Onyura et al., 2017). Reflection on past teaching can help teachers to become aware of the structural rules and processes that determine good teaching practices (Kelchtermans, 2009). This study adds that both individual reflection and reflection with peers and educational experts contributes to university teachers’ well-being through the dimension of self-acceptance.

Second, the well-being dimension of positive relations with others was most strongly associated with the learning community and the formal workshops in the case study. In both activities, the social aspect of professional development was highlighted by the participants, creating opportunities to connect and discuss with each other during the workshops and other community activities during the program, as well as outside of the scheduled program activities. Prior research has already highlighted the positive effects of participating in a learning community and increased interaction and social support between colleagues (Lieberman, 2000; Onyura et al., 2017). Building on Warhurst (2008), formal workshops can also foster positive relations between colleagues when they create knowledge-sharing opportunities. Formal workshops in combination with a safe and open atmosphere within the learning community, participants are more willing to share an issue or solution. Additionally, increased collegiality and a broadened network can also create opportunities for collaboration outside of the professional development program, and in turn also positively affect academic research. Our findings add the notion that university teachers’ well-being is crucially intertwined with the social dimensions of work, which can be facilitated through professional development programs specifically for new teachers.

Third, the well-being dimension of autonomy is not strongly associated with the professional development activities included in the case study. Based on prior research (e.g., Steinert et al., 2010), we expected that the formal workshops would support the feeling of autonomy in teachers through increased knowledge of pedagogical approaches combined with evidence for best practices, so teachers would feel more self-determined about how they regulated their teaching and how they developed educational materials. However, our results suggest that it was not the formal workshops, but rather the interaction with peers in the learning community that supported the feeling of autonomy. Different opinions expressed by other participants in the UTQ program, as well social dynamics, helped participants to regulate teaching behavior from within. Relating these findings to self-determination theory by Deci and Ryan (2002), the contrasting teaching beliefs discussed by participants in the program may have strengthened teachers’ intrinsic motivation to act according to their own teaching beliefs and teaching styles; during the interviews, some teachers expressed that previously, they had often been influenced by the teaching beliefs and practices of senior colleagues, which seemed to set certain departmental norms with regard to teaching. In-depth discussions with peers seem to have strengthened their conviction to resist (some of) these external pressures and act according to their own beliefs, adding to prior research on the link between autonomy, professional development activities, and well-being.

Fourth, formal workshops played the biggest role by far for the well-being dimension of environmental mastery, along with participation in the learning community. We found that formal workshops provide pedagogic tools and structure that support teachers in the ability to control the complex array of teaching activities, in line with findings by Avalos (2011) and Stewart (2014) showing that formal workshops provided clarification and created discussions around teaching policies, practices and administrative procedures. The importance of formal workshops in this study contradicts research stating that formal learning activities are less effective compared to informal learning activities (Kyndt and Baert, 2013), as well as research on the timing of formal workshops that may not align with when teachers need the instruction (Jones and Dexter, 2014). An explanation could be the interactive nature of these workshops in the case setting of the UTQ program, which gave participants the possibility to learn from each other while developing their theoretical knowledge. This synergistic relationship between formal and informal activities was also previously highlighted by Kyndt and Baert (2013). Contrary to prior research findings (e.g., Ilgen et al., 1979; Ashford and Cummings, 1983), feedback was not associated with environmental mastery. In the case setting, interviewees described that they sought and received feedback on specific aspects of their course design and teaching practices, which did not lead to a different use of environmental resources, but rather enhanced their sense of personal growth (see conclusion six below). We conclude that new university teachers’ well-being benefits from formal learning when they are afforded social interaction and informal learning within trainings and workshops.

Fifth, based on prior research by Lieberman (2000), we expected that the learning community would play a significant role in the purpose of teachers, as collaboration in networks can unite faculty members, creating a strong sense of shared purpose, and in turn fostering commitment and engagement. However, in our case study, the UTQ program in its entirety was found to play the most significant role in supporting the teachers’ purpose. The UTQ certificate itself has a certain standing and status within the university as well as nationally, which provided meaning in the present lives of participants, as well as opening up new career opportunities for teachers, as the certificate is often a requirement for promotion (de Jong et al., 2013). Additionally, the workshops were complementary in supporting this belief. The interviews showed that the program transformed the task-oriented view of teaching of many participants to a meaning-oriented view (Stewart, 2014). Adding to these prior findings, the present study shows that participants can derive well-being through purpose by gaining both a meaning-oriented view and a formal symbol of status at the same time, without one crowding out the other.

Finally, all professional development activities supported the participants’ feeling of personal growth. The learning community, formal workshops, and feedback were especially associated with this well-being dimension. Previous studies have shown that professional development programs can lead to positive personal changes in cognitions, beliefs and practice (Stes et al., 2007; Avalos, 2011). Participants in our case study stated that with the knowledge and experience from the formal workshops, they became more open to new experiences and to trying new teaching and assessment methods. This is supported by Butcher and Stoncel (2012), and Stewart (2014), who found sustained personal growth because of a willingness to try new and innovative approaches. Additionally, the results of this study show that personal growth is also supported through discussions in the learning community, woven into the support given in the formal workshops, due to their interactive nature focusing on peer support. Overall, the intellectually challenging environment helped in strengthening interest and energy to recognize their sustained improvement and maturing self-perception as teachers (in line with Onyura et al., 2017). Furthermore, following Locke et al. (1983), role clarity created through workshops and the learning community may have helped to enhance competences and reduce uncertainty regarding goal-related behavior by decreasing uncertainty, leading to feelings of control, as professionals learned which of their decisions led to success and which led to failure. Accordingly, this aligned with teachers’ feeling of sustained growth as a result of the program. Feedback also contributed toward personal growth; however, the perceived fairness of the feedback seems to be crucial, as prior research has shown (Sparr and Sonnentag, 2008). In the current case study, participants focused on feedback on the design and delivery of courses they were currently involved in, leading to hands-on implications and insights. We conclude that teacher well-being is closely related to teachers’ personal growth, specifically for beginning teachers, who benefited from the whole range of professional development activities.

In conclusion, teacher professional development programs can play a significant role for teacher well-being in a context of high workload and pressure, when designed with social interaction, learning community, and reflection in mind.

### Limitations and recommendations for future research

The present study follows an exploratory case-study approach, offering in-depth information on the relationship between teacher professional development activities and teacher well-being at work. Although our sample consisted of teachers from different departments, the sample size was rather small, calling for future research to study the relationships on a larger scale. Furthermore, we studied one specific teacher professional development program in one specific faculty at a Dutch university, limiting the generalizability of our findings. Conducting a comparative study across different institutes of higher education to account for variability in the design and delivery of professional development activities is therefore a critical next step. Specifically, a closer look needs to be taken at coaching and feedback, as in the current study, the support for psychological well-being from coaching and feedback was not conclusive. Other universities may have integrated feedback and coaching to a greater extent in teacher professional development, resulting in additional insights. Furthermore, the perceived value of certain teacher professional development activities for the work well-being of participants might be dependent on the progress of the participant toward completing the program. Participants might evaluate certain professional development activities as more meaningful toward the end of the program or after completion of the program, compared to earlier stages of the program.

A second limitation concerns the heterogeneity of the interviewees. Their demographic and professional characteristics diverged to the extent that one participant had taught at the university for 9 years, but the average was less than half that (3.4 years). Length of employment seems to influence the perceptions of the usefulness of the professional development program; however, we were not able to further explore this link given the present sample. The results for the formal workshops in particular indicated that newcomers perceived the program as crucial for their professional development, compared to the more negative perceptions of teachers with longer employment. The differences in experience and knowledge were laid bare in the workshops, where certain regulations were new to some, but repetitive for others. It would be interesting to explore how professional development programs can remain relevant for KSAs and well-being for more experienced teachers as well. Further avenues for research should therefore aim to compile a more homogenous sample or focus on a comparative study between teachers with shorter and longer employment.

Third, due to the COVID-19 regulations in the Netherlands at the time of data collection, it was impossible to conduct the interviews in person. Although a video call was sufficient to establish a relationship, there were two interviewees who preferred an audio call only. Additionally, due to weak internet connections, certain questions or answers were repeated, and this detracted from the sincerity of the initial story. Nevertheless, the qualitative research design allowed for deep understanding of the relationship between teacher professional development activities and teacher well-being. Based on our exploratory study, future research should establish causal relationships through large-scale quantitative research.

### Implications for practice

The results presented in this study are relevant for educational developers who seek to pay explicit attention toward supporting psychological well-being of teachers. To address well-being, universities should include different types of activities that lead to changes in teachers’ professional practice as well as in their thinking about that practice—our study shows that including specific professional development activities in such a program can be effective for targeting specific dimensions of well-being at work.

First, creating possibilities for social exchange between participants is crucial for almost all dimensions of well-being. Creating possibilities for teachers to discuss their teaching experiences and learn from each other can increase their sense of environmental mastery and fosters collegiality. Implementing a learning community for teachers can help them to resist social pressures and become more self-determined in their teaching. The learning community also provides a platform for teachers to approach each other on a deeper level with the collective purpose of enhancing student learning (Vescio et al., 2008). In addition, it can create a sense of shared purpose across departments. Our participants highlighted that this social exchange can be part of both formal and informal development activities, as long as formal workshops are designed with interaction, peer learning, and social connection in mind. Social exchange was furthermore advanced through organizing participants in cohorts, and through a shared start and end point in the program.

Furthermore, new teachers in particular are often struggling with insecurities regarding their teaching role (Morris and Usher, 2011; Butcher and Stoncel, 2012). Creating various possibilities for in-depth reflection on their own teaching practices and beliefs can help teachers to identify improvement points, but also to accept their professional strengths and weaknesses. This self-knowledge facilitates course design and delivery that best fit their teaching style, fostering well-being over time. In addition, in light of the research–teaching nexus, this self-awareness can lead to increased clarity about those roles and the identification of an optimal proportion of teaching activities (Norton et al., 2010). In the current UTQ program, reflection is facilitated and stimulated in three main ways: through structured individual intake, ongoing group reflection combined with theoretical knowledge in the workshops, and a written reflective portfolio that is assessed formatively and summatively by a teaching expert. This portfolio provides a scaffolding structure for reflection through specifying different roles and competences participants develop during the UTQ program, and encourages participants to seek external feedback and to provide examples for more in-depth reflection.

Finally, the importance of a teacher professional development program for teachers’ purpose in life depends to a large extent on the importance this program is given within the institution, as well as on a national level. Although most participants in our study seemed to be intrinsically motivated to improve their teaching practices, participation in the program is mandatory, in many cases, for promotion. Even very intrinsically motivated teachers often do not prioritize their professional development in this regard, as departmental expectations conflict with their own academic identity (Van Lankveld et al., 2017). Embedding teacher professional development in institutional performance evaluation practices can help to foster continual teacher professional development. At the same time, the design of teacher professional development programs needs to account for potential trade-offs between intrinsic and extrinsic motivation, regarding both the development of KSAs and well-being. Initiatives to support teacher well-being and teacher professional development initiatives are usually seen as separate from one another, we propose that by designing teacher professional development programs for relatedness (through social relationships and learning communities), autonomy (through interactive workshops and community), and competence (through reflection, workshops, and feedback), new university teachers’ well-being can be actively supported.

## Data availability statement

The raw data supporting the conclusions of this article will be made available by the authors, without undue reservation.

## Ethics statement

Ethical review and approval was not required for the study on human participants in accordance with the local legislation and institutional requirements. The patients/participants provided their written informed consent to participate in this study.

## Author contributions

IG: conceptualization. IG and MN: methodology, formal analysis, and investigation. All authors wrote and edited the manuscript.
